# The seed oil of *Paeonia ludlowii* ameliorates Aβ25‐35‐induced Alzheimer's disease in rats

**DOI:** 10.1002/fsn3.2102

**Published:** 2021-03-30

**Authors:** Ya‐Zhou Lu, Chao‐Qi Zhang, Ben‐Xia Yu, Er‐Hao Zhang, Hong Quan, Xiu Yin, Hao Cai, Fang Yuan, Lian‐Qiang Li, Yuan‐Jiang Xu, Yan‐Jie Su, Ya‐Jing Xing, Zhi‐Hua Liao, Xiao‐Zhong Lan

**Affiliations:** ^1^ TAAHC‐SWU Medicinal Plant Joint R&D Centre Tibetan Collaborative Innovation Centre of Agricultural and Animal Husbandry Resources Food Science College Tibet Agricultural and Animal Husbandry University Nyingchi China; ^2^ Key Laboratory of Forest Ecology in Tibet Plateau (Tibet Agricultural & Animal Husbandry University) Ministry of Education Nyingchi China; ^3^ Chongqing Institute of Traditional Chinese Medicine Chongqing China; ^4^ SWU‐TAAHC Medicinal Plant Joint R&D Centre Southwest University Chongqing China

**Keywords:** Alzheimer's disease, apoptosis, cognitive function, neuronal damage, *Paeonia ludlowii* seed oil

## Abstract

*Paeonia ludlowii*, a plant of the Paeoniaceae family, has abundant genetic diversity in different populations, and the seed oil can be used in a diverse number of activities. However, its neuroprotective effect is not clear. We investigated the memory‐improving effects and associated mechanisms of *Paeonia ludlowii* seed oil (PLSO) on amyloid beta (Aβ)25–35‐induced Alzheimer's disease (AD) in rats. The Morris water maze test was undertaken, and subsequently, the content of malondialdehyde (MDA), superoxide dismutase (SOD), glutathione (GSH), and acetylcholinesterase (ACHE) in the hippocampus was detected by biochemical analyses. To further study PLSO, we examined the pathologic structure and apoptosis of hippocampal tissue by staining. Immunohistochemical analysis was used to detect expression of IBA‐1 and GFAP in the hippocampus. Detection of proinflammatory factors was achieved by reverse transcription–quantitative polymerase chain reaction and Western blotting. High‐dose PLSO inhibited expression of GFAP and IBA‐1. We demonstrated that high‐dose PLSO can regulate activation of glial cells and mediate apoptosis of hippocampal cells, and significantly improve learning and memory deficits in AD rats. PLSO could be developed as a nutritional supplement and sold as a drug for AD prevention and/or treatment.

## Graphical abstract

It has broad research and application value in food, healthcare products, medicine, and other aspects, which gives AD patients an additional healthy choice in their lives.

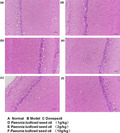

## 1 INTRODUCTION

Alzheimer's disease (AD) dementia refers to a particular onset and course of cognitive and functional decline associated with age together with a particular neuropathology (Soria Lopez et al., 2019). It is the most common cause of dementia worldwide and reported to be the sixth leading cause of death for citizens in the USA (Masters et al., 2015). Aging is considered to be the main risk factor for AD. The main manifestations of AD are memory loss, cognitive impairment, and personality change (Graham et al., 2017). The pathologic features of AD are the accumulation of “senile plaques” and intracellular neurofibrillary tangles, accompanied by the loss of synapses and neurons, leading to cognitive dysfunction and, eventually, dementia (Congdon et al., 2018; Kimura et al., 2016).

Five drugs have been approved for AD treatment. Four are acetylcholinesterase (ACHE) inhibitors (donepezil, tacrine, rivastigmine, and galantamine), and one is an antagonist of N‐methyl‐D‐aspartate receptors (memantine). Donepezil (Aricept^®^) is a centrally acting reversible ACHE inhibitor. It is first‐choice AD treatment recommended by the National Institute for Health and Care Excellence in the UK. However, like other AD drugs, donepezil improves the cognition and behavior of AD patients without slowing down AD progression (Lee et al., 2015). In addition, donepezil has several side effects, such as loss of appetite, gastrointestinal upset, sleeping difficulty, and muscle cramp (Querfurth et al., 2010). Moreover, due to poor efficacy or severe adverse reactions, few clinical trials of AD drugs have been successful (Godyń et al., 2016). A new drug for AD treatment has not been approved since 2003. In spite of this grave situation, no effective treatments are available for AD (Scheltens et al., 2010). There is an urgent need to find and develop novel anti‐AD drugs or nutritional supplements that are more effective and/or less toxic.

Natural products have a significant role in the prevention of disease and boosting of health in humans (Rehman et al., 2019). Some scholars have suggested that various oils and nutrients exhibit preventative effects against AD, for example, fish oil (Xiang et al., 2016; Xie et al., 2020), walnut oil (Liao et al., 2020), and Rhodiola rosea (Vasileva et al., 2016). The roots of Paeonia ludlowii have been used in Tibetan folk medicine for ~100 years for treatment of diseases (gynecological, skin, cardiovascular, cerebrovascular) (Jiang et al., 2017). Some studies have been carried out on its chemical composition, endophytic bacteria, and habitat profile (Jiang et al., 2016; Lu et al., 2020; Yang et al., 2007; Zeng et al., 2015). However, studies on improvement of memory impairment using *Paeonia ludlowii* seed oil (PLSO) have not been carried out. Based on previous study from our research group (zhang et al., 2020), it is speculated that the PLSO may have the effect of enhancing memory. This study addressed to investigate whether PLSO can improve ameliorating learning and memory impairment in Aβ‐induced rat model of AD, as well as to clarify the possible mechanism briefly underlying the neuroprotective effect of PLSO in ameliorating.

## 2 MATERIALS AND METHODS

### 2.1 Ethical approval of the study protocol

This study was carried out in strict accordance with the recommendations provided in the *Guide for the Care and Use of Laboratory Animals* of the US National Institutes of Health (Bethesda, MD, USA). Experimental protocols were approved by the Institute of Experimental Zoology, Chongqing Academy of Chinese Materia Medica (permit number SYXK [Chongqing] 2017–0003) in Chongqing, China.

### 2.2 Reagents and chemicals

Donepezil hydrochloride tablets (lot: 110822A) were purchased from Eisai China and prepared in a mixed suspension at 0.1 mg/ml. Aβ25‐35 (lot: Y‐0044) was obtained from Bioss Biotech. Streptavidin–biotin complex and terminal deoxynucleotidyl transferase dUTP nick‐end labeling (TUNEL) apoptosis detection kits were purchased from Boster Biotech. A real‐time reverse transcription–quantitative polymerase chain reaction (RT‐qPCR) kit was obtained from TransGen Biotech. Antibodies against Bcl‐2 Bax, and β‐actin were purchased from Bio‐Rad Laboratories. A brain stereotaxic apparatus (ZH‐LAN‐STAR, C type) was obtained from Huaibei Zhenhua Biological Equipment. An inverted microscope (S70) was purchased from Leica. A ChemiDoc XRS gel imaging and analysis system was obtained from Bio‐Rad Laboratories. An SP‐9000 immunohistochemistry kit was purchased from Beijing ZSGB Biological Engineering. PCR amplification instrument (TC‐512) was from Techen. Detection kits for malondialdehyde (MDA), superoxide dismutase (SOD), glutathione (GSH) and ACHE were purchased from Nanjing Jiancheng Bioengineering Research Institute. Rabbit anti‐GFAP antibody (lot: 16825–1‐AP) was obtained from Proteintech. Rabbit anti‐IBA1 antibody (lot: bs‐1363R) was from Bioss Biotech.

### 2.3 Plant material and extraction

*Paeonia ludlowii* samples were collected from the Himalayan Hengduan Mountains in MiRui Township (29º32′43.96″N, 94º38′33.03″E) in Nyingchi City (Tibet, China) in October 2018. They were identified by Professor Xiaozhong Lan at the School of Food Science within Tibet Agriculture and Animal Husbandry University (Nyingchi City). The seed pretreatment and oil extraction process of *Paeonia ludlowii was carried out as follows*: first of all, the full, normal size, no mechanical damage, and no mildew *Paeonia ludlowii* seeds were picked out, then manually its black outer kernel coats were removed to obtain *Paeonia ludlowii* kernels, subsequently, which was placed in a drying oven at 65°C to a constant quality, and the press machine was set in motion, then the *Paeonia ludlowii* seed kernel was added into the feeder, preheated for 6 min, scent was set and pressed, and after pressing and extraction, the crude oil in the collecting bottle was filtered to obtain the *Paeonia ludlowii* seed oil.

### 2.4 Experimental animals

Specific‐pathogen‐free male Sprague Dawley rats (6 months; 180–220 g) were purchased from Shanghai SIPPR‐BK Laboratory Animals (certificate number 20170005014637). Animals were allowed to acclimatize for 1 week in an environmentally controlled breeding room at room temperature (25 ± 1°C) and a 12‐hr light/dark cycle. Animals had access to food and water ad libitum.

### 2.5 Experimental design

Rats were divided randomly into six groups of six: control; model; positive (donepezil 0.42 mg/kg); low‐dose PLSO (1 g/kg); medium‐dose PLSO (2 g/kg); and high‐dose PLSO (10 g/kg).

Except for the control group, the AD model was established in all groups. Anesthesia was induced by 1% pentobarbital sodium (40 μg/g, i.p.). Then, rats were fixed on the brain stereotaxic instrument. According to the stereotaxic map of the rat brain, the anterior fontanel was the starting point, 3.5 mm behind the bregma was the puncture point, and the right side of the midline was opened 2 mm, and a posterior dental drill was used to open the skull (AP = −3.5 mm, Ml = 2.0 mm, DV = 3.0 mm). Then, 10 μg (1 μl) of Aβ1‐40 was injected at both sides of the CA1 area of the hippocampus at a slow and uniform rate, the needle was retained for 5 min, and the wound was closed after needle withdrawal. Drug administration started 4 days after the model had been created. Rats were gavaged according to each dose of the drug, once a day for 2 weeks; the control group and model group were given physiologic (0.9%) saline.

### 2.6 Morris water maze test (MWMT)

The spatial learning was investigated using the Morris water maze. The general testing procedure was described previously (Li et al., 2007). Rats were removed from cages and placed rapidly into the water maze. When the rat entered the water through the monitoring system, the button on the video recorder was pressed immediately. When the two cooperate, the rat pressed the button when entered the water. The recording time was set to 90 s. When the rat arrived at the station platform and stayed there for 30 s, this was regarded as “finding the station,” and video recording stopped automatically. If the rat did not swim on to the platform within 90 s, a wooden stick was used to guide the rat to the platform immediately. The rat was removed after becoming familiar with the surrounding environment on the platform for 10 s; if the rat arrived on the platform or stayed for a specified time, he was taken away again after becoming familiar with the environment for 10 s. After removing the rat from the water maze, he was dried with a towel. After completing each set of experiments, the next round of experiments was carried out at 30‐min intervals. Four rounds of experiments were completed. Positioning navigation experiment was used for 4 days. On day 5, remove the platform, select the opposite quadrant of the quadrant where the platform is located as the water entry point, only do it once, and the recording time is 60 s.

### 2.7 Biochemical test

Biochemical test was performed according to protocol published in previous study (Engvall et al., 1971). Detection kits for MDA, SOD, GSH, and ACHE were operated according to the manufacturer's (Nanjing Jiancheng Bioengineering Research Institute) instructions. The content of each compound was calculated according to the formula.

### 2.8 Histopathology of the hippocampal neurons of rats

Anesthesia was induced with pentobarbital sodium (1.0%, i.p.). Brains were removed from anesthetized rats and weighed. Brain tissue was fixed in 10% neutral buffered formalin, dehydrated, and embedded in paraffin. Coronal sections were obtained using a microtome, followed by staining with hematoxylin and eosin (H&E) for pathology studies under light microscopy (200×), according to protocol published in previous study (Alghamdi et al., 2018).

### 2.9 Neuron apoptosis by the TUNEL assay

The apoptotic study was carried out with TUNEL analysis as previously described (Fang et al., 2005). TUNEL staining was used to assess apoptosis. Tissue blocks were placed in 10% formalin for 48 hr. After washing with phosphate‐buffered saline (PBS), staining with 3,3‐diaminobenzidine was done for 3–10 min. This action was followed by rinsing with tap water, counterstaining with hematoxylin, and differentiation with 1% hydrochloric acid in alcohol. After observation under a microscope, the degree of staining was controlled. Dehydration with different concentrations of alcohol was undertaken for 3 min each time. Sealing with neutral gum was completed. Images were captured under an inverted microscope (S70; Leica) and the apoptosis rate was calculated.

### 2.10 Immunohistochemical (IHC) analyses

The reactive microglia and astrocytes in the brain samples were visualized by immunohistochemistry (IHC) that was performed as described in the previous study with slight modifications (Delcambre et al., 2016). Tissue blocks were placed in 10% formalin for 48 hr. Fixed tissue was washed with running water to remove residual fixative and impurities. Ethanol of different concentrations enabled dehydration in a step‐by‐step manner. Blocks were cleared, soaked in wax, and embedded in paraffin. Slides were placed in a constant‐temperature oven at 65°C for 30 min. Then, they were placed in xylene I solution and allowed to soak for 15 min. Next, they were placed in xylene II solution for 15 min to dewax and rehydrate. Sodium citrate buffer solution (0.01 M) was employed for high‐pressure heat repair for 15 min. After natural cooling, slides were washed thrice with PBS (0.02 M) for 3 min each time. Microwave treatment in sodium citrate (0.01 mol/L) for 15 min was carried out. EDTA (pH 8.0) or 0.01 M sodium citrate buffer solution (pH 6.0) was added to boiling water for high‐pressure heat repair. Next, slides were placed in 3% H_2_O_2_ and allowed to incubate for 10 min in a wet box to eliminate derived peroxidase activity. Then, sections were incubated with primary antibody (CHOP; 1:100 dilution; GRP78, 1:50; caspase‐3, 1:50; Proteintech) at 4°C overnight. Finally, sections were incubated with horseradish peroxidase‐conjugated secondary antibodies (Santa Cruz Biotechnology) and counterstained with hematoxylin. All tests were carried out on at least three sections of each sample. Images were recorded through a microscope, the relevant parts of the sample observed, and the positive area calculated.

### 2.11 RT‐qPCR

Reverse transcription–quantitative polymerase chain reaction was carried out according to protocol published in previous study (Yang et al., 2017). Total RNA was extracted with TRIzol^®^ Reagent (Invitrogen) from hippocampal tissues, reverse‐transcribed, and amplified by RT‐qPCR according to manufacturer instructions. The primers used for qRT‐PCR are shown in Table 1. Glyceraldehyde 3‐phosphate dehydrogenase (GAPDH) served as the housekeeping gene. Experiments were carried out in triplicate.

**TABLE 1:** The primer sequence, PCR target, and cycle for mRNA of TNF‐α, IL‐1β, and IL‐6

mRNA	Primer sequence PCR	Target (bp)	Cycle
TNF‐α	F: 5′TGGCGTGTTCATCCGTTC−3′	199	40
	R: 5′CTACTTCAGCGTCTCGTGTG−3′		
IL−1β	F: 5′ATCTCACAGCAGCATCTC−3′	189	40
	R: 5′TAGCAGGTCGTCATCATC−3′		
IL−6	F:5′CACCAGGAACGAAAGTCAACTC−3′	112	40
	R:5′GTGGCTGTCAACAACATCAGTC−3′		

#### 2.11.1 Western blotting

Western blotting was performed according to protocol published in previous study (Xia et al., 2017). Total proteins were isolated using a protein extraction kit (Bio‐Rad Laboratories) according to standard protocols. The antibodies used were tumor necrosis factor (TNF‐)α (1:600 dilution), interleukin (IL‐)1β (1:1,000), IL‐6 (1:1,000), Bax (1:500), Bcl‐2 (1:500), p65 (1:1,000), phosphorylated (p)‐p65 (1:1,000), and GAPDH (1:2000), all of which were from Cell Signaling Technology. Horseradish peroxidase‐conjugated secondary antibodies (Santa Cruz Biotechnology) were used, and protein bands were detected using an enhanced chemiluminescence system. GAPDH was used as the housekeeping gene. Experiments were carried out in triplicate.

### 2.12 Statistical analyses

Data are the mean ± standard deviation of at least three independent experiments. Differences between groups were calculated using one‐way analysis of variance (ANOVA). Statistical analyses were undertaken using SPSS v19.0 (IBM). Differences between experimental groups were considered significant at *p < *.05, very significant at *p < *.01, and extremely significant at *p < *.001.

## 3 RESULTS

### 3.1 PLSO improved the escape latency and dwelling time on platform quadrants in AD rats

**FIGURE 1:**
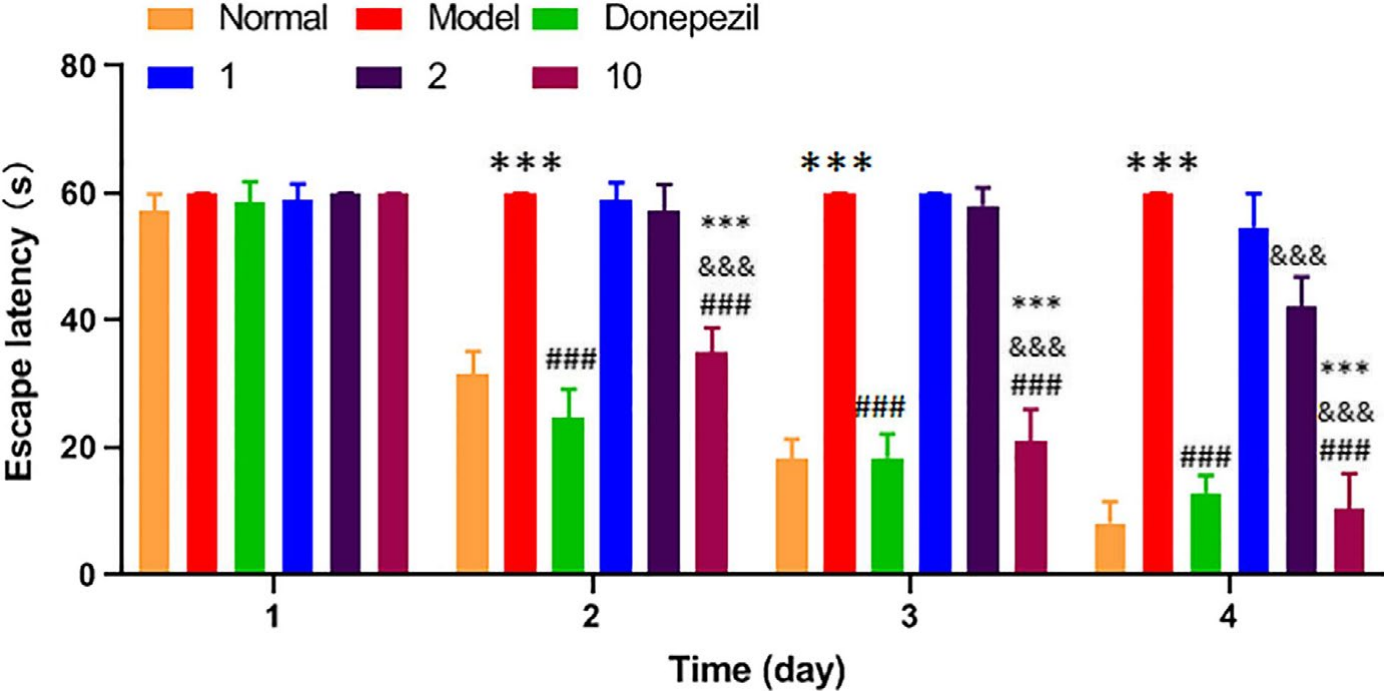
Escape latency. Results are representative of at least three independent experiments and expressed as the mean ± *SD*. ***p < .001 versus control group; ^###^ *p* < .001 versus model group; ^&&&^ *p* < .001 versus medium‐dose PLSO group; and ^❉❉❉^ *p* < .001 versus low‐dose PLSO group

We administered the MWMT to evaluate the influence of PLSO on the behavioral memory of AD rats. With increasing training time and time, the escape latency of the model group did not change significantly (Figure 1). For the AD model group, compared with the control group, from day 2 to day 4, the escape latency was extremely significantly longer (*p* < .001). Compared with the model group, the positive group (donepezil) had an extremely significantly shorter escape latency from day 2 to day 4 (*p* < .001). The low‐dose PLSO group showed no significant difference compared with that of the model group (*p* > .05). On the fourth day of training, the middle‐dose PLSO group was extremely significantly different from the model group (*p* < .001). The high‐dose PLSO group, compared with the model group, had an extremely significantly shorter escape latency from day 2 to day 4 (*p* < .001). In the medium‐dose PLSO group, compared with the low‐dose PLSO group, on day‐4, the escape latency was extremely significantly different (*p* < .001). Compared with the low‐dose PLSO group, the high‐dose PLSO group had an extremely significantly shorter escape latency (*p* < .001). Compared with the medium‐dose PLSO group, the high‐dose PLSO group had an extremely significantly shorter escape latency (*p* < .001).

**FIGURE 2:**
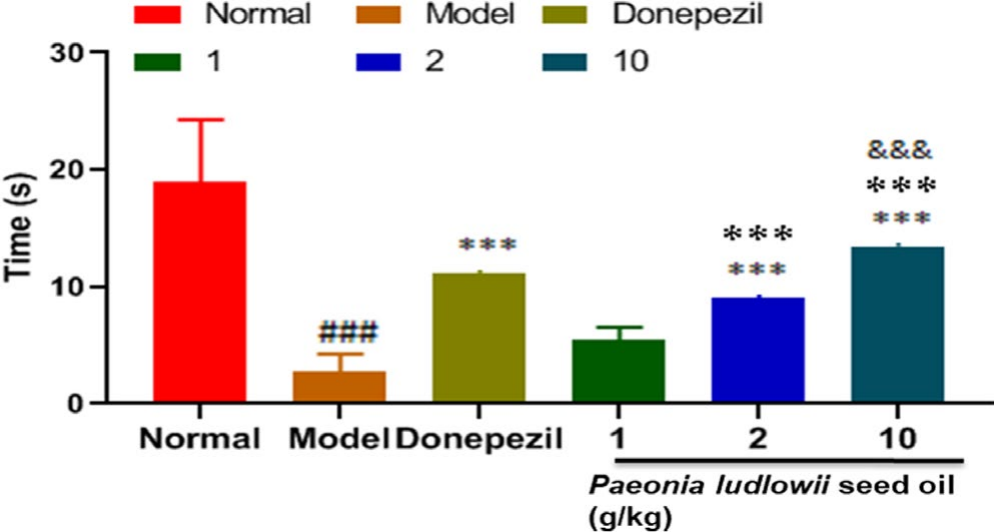
Dwelling time on platform quadrants. Results are representative of at least three independent experiments and expressed as the mean ± *SD*. ***p < .001 versus control group; ^###^ *p* < .001 versus model group; ^&&&^ *p* < .001 versus medium‐dose PLSO group; and ^❉❉❉^ *p* < .001 versus low‐dose PLSO group

Among the MWMT, positioning and navigation experiments, the activity time in the quadrant where the platform was located is shown in Figure 2. Compared with the control group, the activity time of the model group was extremely significantly shorter (*p* < .001). Compared with the model group, the activity time of the positive group was extremely significantly longer (*p* < .001). Compared with the model group, the activity time of the low‐dose PLSO group was not significantly different (*p* < .05); medium‐dose group was extremely significantly longer (*p* < .001); and high‐dose group was extremely significantly longer (*p* < .001). Compared with the low‐dose PLSO group, the activity time of the medium‐dose group was extremely significantly longer (*p* < .001); high‐dose PLSO group was extremely significantly longer (*p* < .001). Compared with the medium‐dose PLSO group, the activity time of the high‐dose group was extremely significantly longer (*p* < .001).

The results stated above suggest that the escape latency of rats in the high‐dose PLSO group (10 g/kg) was shorter, which reflects their better memory. After platform removal, rats were more active in the quadrant where the platform was located, which also reflected their memory. In summary, the high‐dose PLSO group (10 g/kg) could improve the memory of rats significantly and improve AD.

### 3.2 PLSO reversed the pathologic morphology of hippocampal tissue in AD rats

**FIGURE 3:**
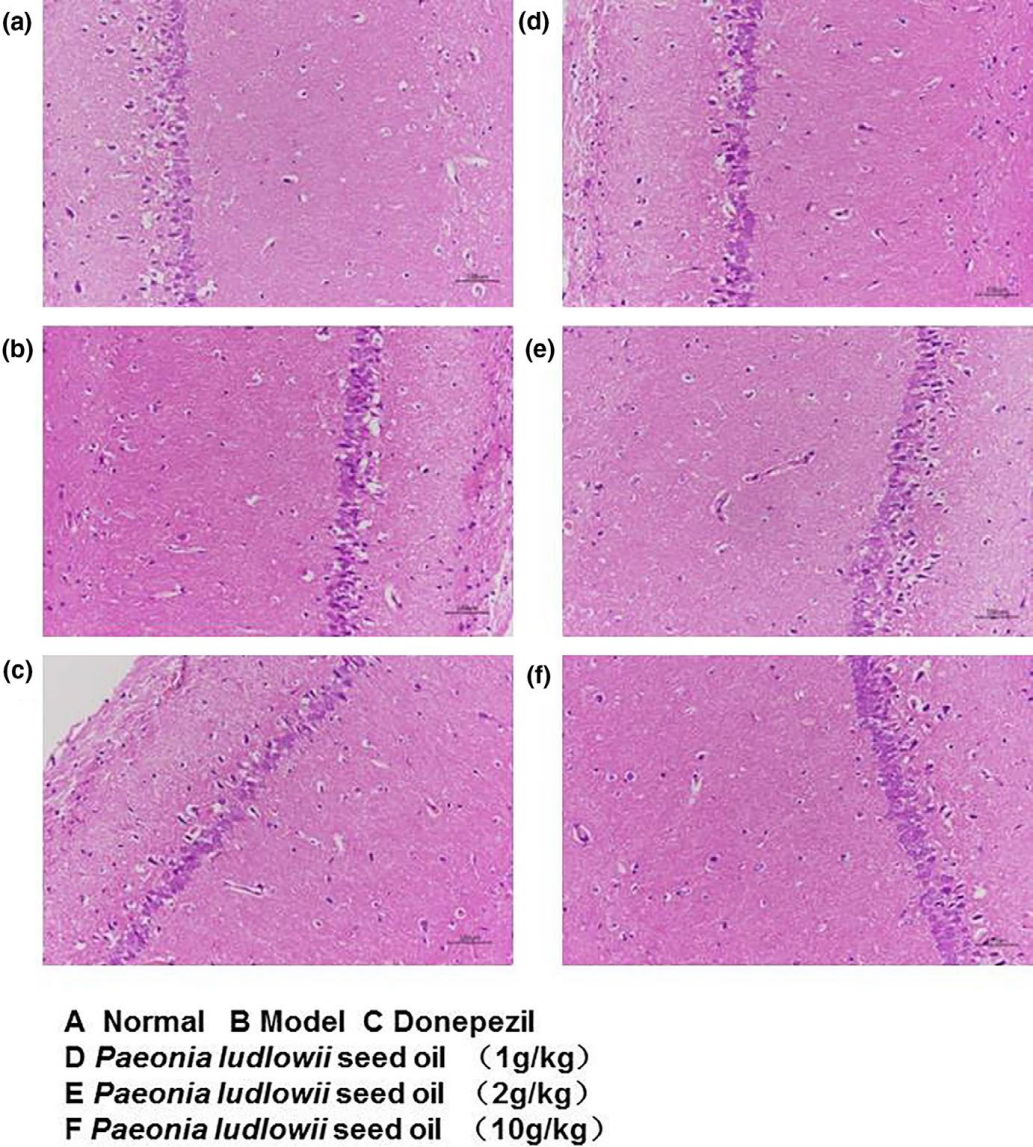
Hippocampus of rat tissue (H&E staining). (a): Control group; (b): model group; (c): donepezil group; (d): PLSO group (1 g/kg); (e): PLSO group (2 g/kg); and (f): PLSO group (10 g/kg)

Next, we undertook pathologic H&E staining on the CA1 area of hippocampal tissue (Figure 3). Figure 3a shows the characteristic preserved normal histologic features of neuronal cells in a control rat treated with physiologic saline. Cells are arranged closely and in an orderly manner. A lightly stained large and circular nucleus and a clear nucleolus are observed; there are no obvious signs of cytoplasmic/nuclear condensation or other neuronal degeneration and damage.

However, the neuronal cells in AD rats treated with physiologic saline (Figure 3b) displayed a loose arrangement with reduced numbers, irregular morphology, decreased cell volume from cytoplasm condensation, and karyopyknosis. Interestingly, the high‐dose PLSO group (10 g/kg) revealed a significant relieving effect.

### 3.3 PLSO alleviated inflammation in hippocampal tissue in AD rats

**FIGURE 4:**
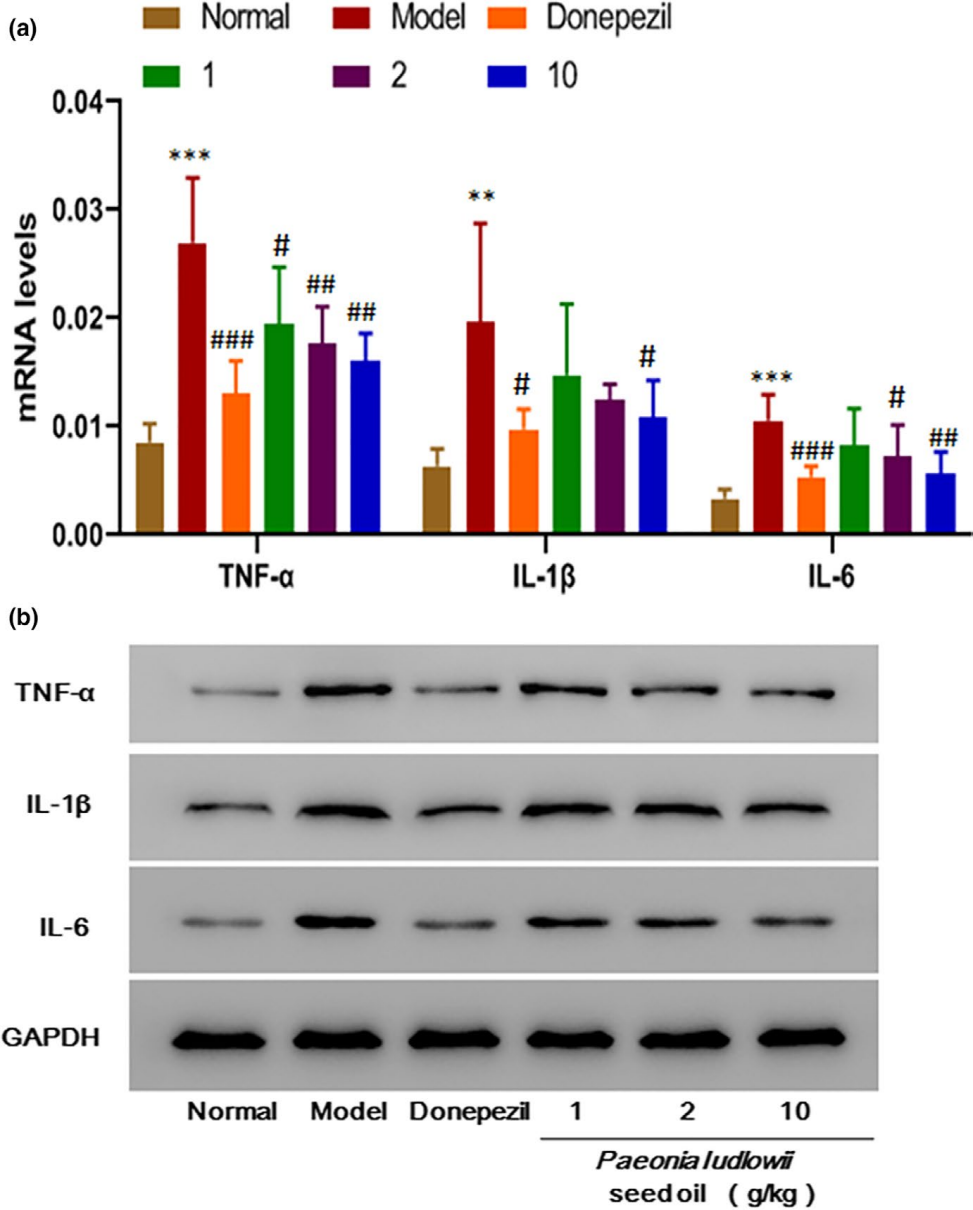
Expression of proinflammatory factors. (a): Results are representative of at least three independent experiments and expressed as the mean ± *SD*. Model group versus control group, ***p < .001; positive group versus model group, ^###^ *p* < .001; medium‐dose PLSO group versus model group, ^#^ *p* < .05; and high‐dose PLSO group versus model group, ^##^ *p* < .01. (b): Effect of PLSO on protein expression of TNF‐α, IL‐1β and IL‐6

We also conducted RT‐qPCR and Western blotting of proinflammatory factors (TNF‐α, IL‐1β, IL‐6) in hippocampal tissue (Figure 4).

#### 3.3.1 TNF‐α

The model group had extremely significantly higher expression than that of the normal group (*p* < .001). The positive group had extremely significantly lower expression than that of the model group (*p* < .001). The low‐dose group of PLSO had significantly lower expression than that of the model group (*p* < .05). The medium‐dose PLSO group had lower expression than that of the model group, and the difference was very significant (*p* < .01). Compared with the model group, the high‐dose PLSO group had lower expression, and the difference was very significant (*p* < .01).

#### 3.3.2 IL‐1β

The model group had higher expression than that of the control group, and the difference was very significant (*p* < .01). Compared with the model group, expression in the positive control group was lower, and the difference was significant (*p* < .05). The low‐dose PLSO group and the medium‐dose PLSO group showed no difference in expression compared with that of the model group (*p* > .05). The high‐dose PLSO group showed lower expression than that of the model group, and the difference was significant (*p* < .05).

#### 3.3.3 IL‐6

The model group showed higher expression than that of the control group, and the difference was extremely significant (*p* < .001). The positive group showed lower expression than that of the model group, and the difference was extremely significant (*p* < .001). The medium‐dose PLSO group had lower expression than that of the model group, and the difference was significant (*p* < .05). The high‐dose PLSO group had lower expression than that of the model group, and the difference was very significant (*p* < .01).

The results stated above suggest that expression of proinflammatory factors was low in the high‐dose PLSO group.

### 3.4 PLSO alleviated oxidant stress of hippocampal tissue in AD rats

**FIGURE 5:**
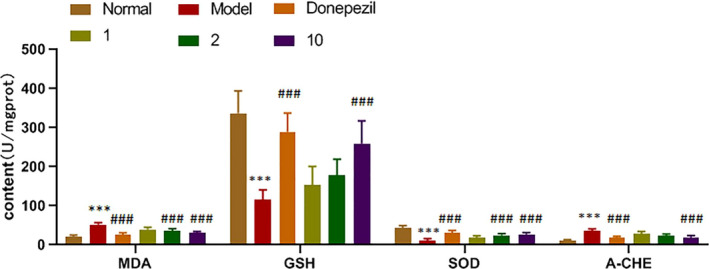
Expression of antioxidants. Results are representative of at least three independent experiments and expressed as the mean ± *SD*. Model group versus control group, ***p < .001; and positive group versus model group, ^###^ *p* < .001

The action of proinflammatory factors affects the antioxidant capacity of cells. Biochemical methods were used to measure expression of the oxidation‐related moieties MDA, SOD, GSH and ACHE (Figure 5).

#### 3.4.1 MDA

Compared with the control group, the model group had higher content, and the difference was extremely significant (*p* < .001). Compared with the model group, the positive group, medium‐dose PLSO group, and high‐dose PLSO group had lower content, and the difference was extremely significant (*p < *.001).

#### 3.4.2 GSH

Compared with the control group, the model group had lower content, and the difference was extremely significant (*p < *.001). Compared with the model group, the positive group and high‐dose PLSO group had higher content, and the difference was extremely significant (*p < *.001).

#### 3.4.3 SOD

Compared with the control group, the model group had lower content, and the difference was extremely significant (*p < *.001). Compared with the model group, the positive group and high‐dose PLSO group had higher content, and the difference was extremely significant (*p < *.001).

#### 3.4.4 ACHE

Compared with the control group, the model group had higher content, and the difference was extremely significant (*p < *.001). Compared with the model group, the positive group and high‐dose PLSO group had lower content, and the difference was extremely significant (*p < *.001).

These results suggested that PLSO could inhibit activation of microglia and astrocytes, inhibit expression of proinflammatory factors, and, thus, affect the antioxidant capacity of cells.

### 3.5 PLSO‐activated glial cells in the hippocampal tissue of AD rats

**FIGURE 6:**
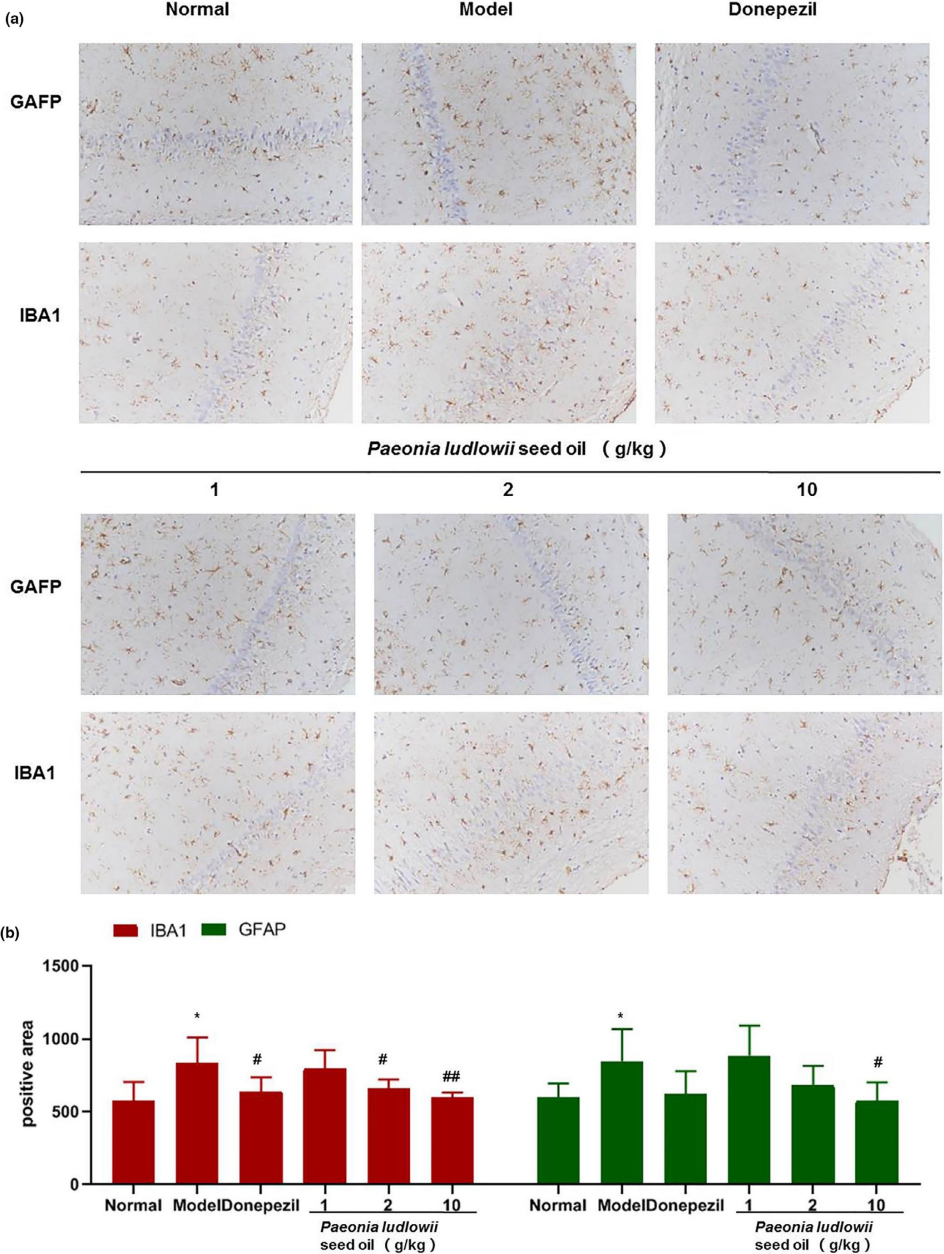
Immunohistochemical analyses (a) and (b) positive areas of GFAP and IBA1. Results are representative of at least three independent experiments and expressed as the mean ± *SD*. Model group versus control group, *p < .05; positive group versus model group, ^###^ *p* < .001; medium‐dose PLSO group versus model group, ^#^ *p* < .05; and high‐dose PLSO group versus model group, ^##^ *p* < .01

To study the specific effect of PLSO on AD, we tested microglia and astrocytes in hippocampal tissues. Expression of the microglia marker, IBA1, and astrocyte marker, GAFP, was measured by IHC analyses (Figure 6b).

For GAFP, compared with the control group, the model group had significantly higher expression (*p* < .05). The high‐dose PLSO group (10 g/kg) had significantly lower expression than that in the model group (*p* < .05).

For IBA1, the model group had significantly higher expression than that of the control group (*p* < .05). The medium‐dose group (2 g/kg) of PLSO had significantly lower expression than that of the model group (*p* < .05). The high‐dose group of PLSO (10 g/kg) had very significantly lower expression than that of the model group (*p* < .01).

### 3.6 PLSO alleviated inflammation via inhibition of the nuclear factor‐kappa B (NF‐κB) signaling pathway

**FIGURE 7:**
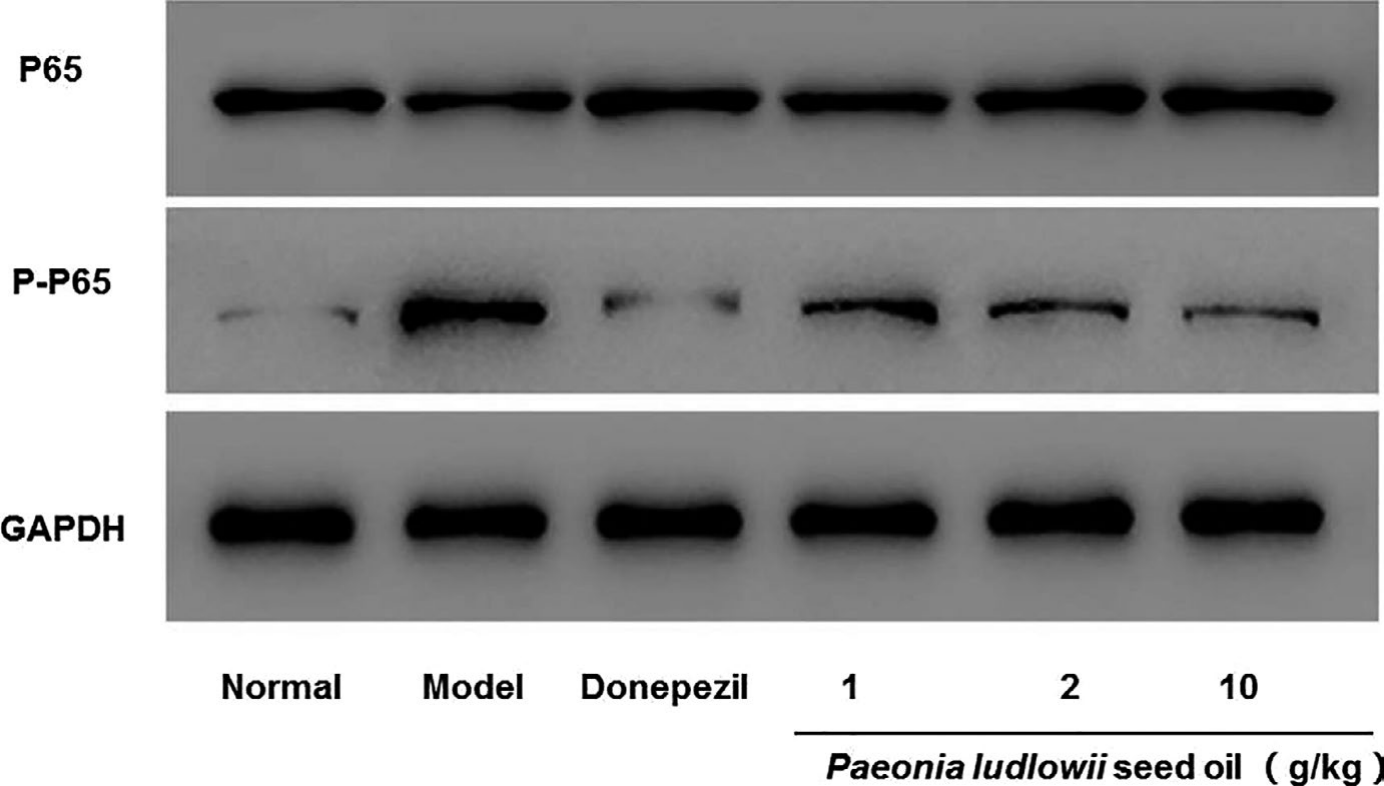
Effect of PLSO on the protein expression of proinflammatory factors and p65 and p‐p65

*Paeonia ludlowii* seed oil mediates expression of proinflammatory factors and then activates the NF‐κB signaling pathway, thereby affecting apoptosis and reducing neuronal‐cell damage. To further study PLSO, Western blotting was done for p65 (one of the five components of the NF‐κB signaling pathway) and p‐p65 in the NF‐κB signaling pathway (Figure 7).

### 3.7 PLSO inhibited apoptosis

Expression of proinflammatory factors was low in the high‐dose group of PLSO. Compared with the model group, expression of p65 and Bax was lower in the high‐dose PLSO group, whereas Bcl‐2 expression was higher in the high‐dose group of PLSO.

**FIGURE 8:**
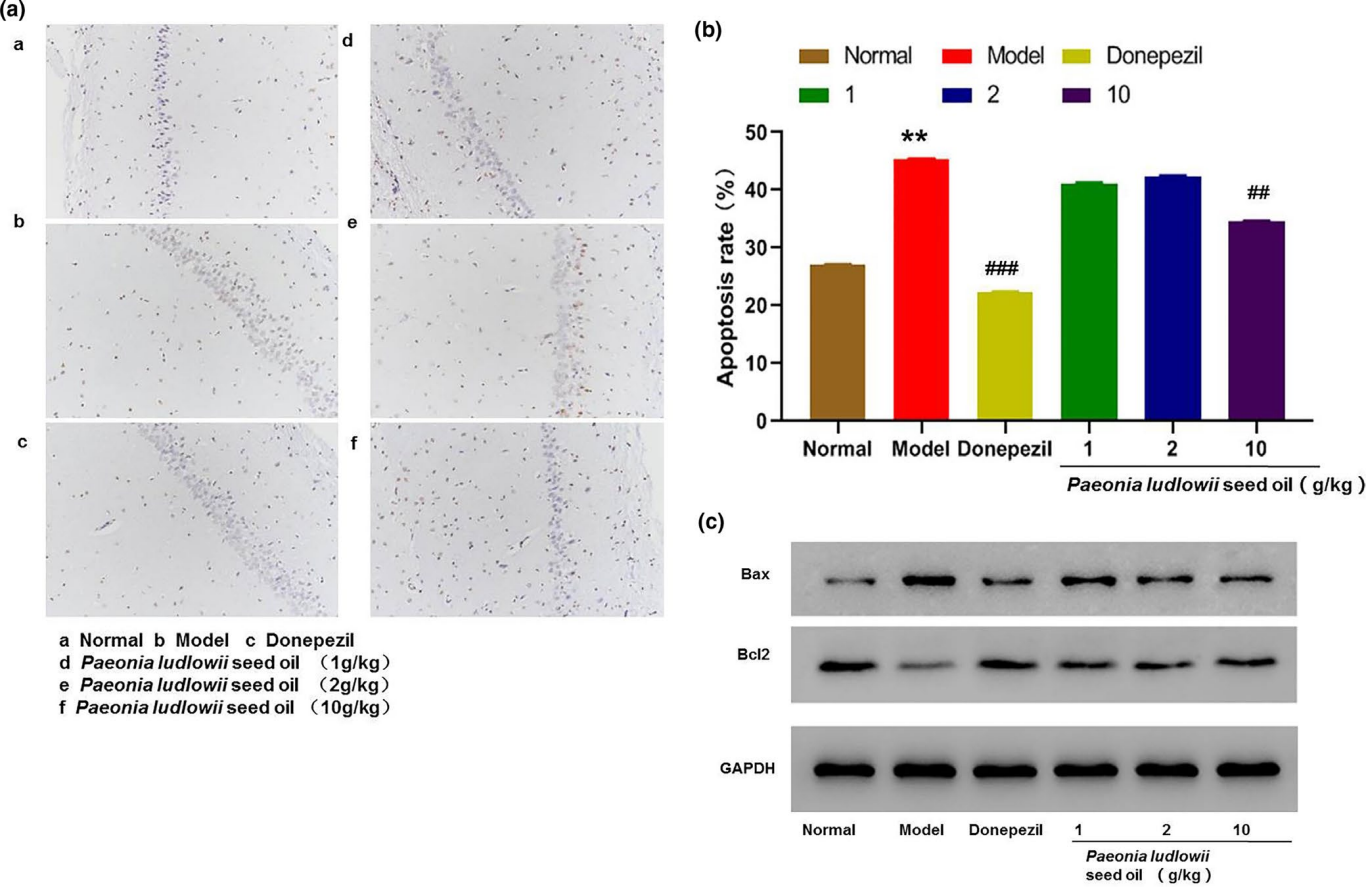
Apoptosis rate. (a): Apoptosis result using the TUNEL assay. a: Control group; b: model group; c: donepezil group; d: PLSO (1 g/kg) group; e: PLSO (2 g/kg) group; and f: PLSO (10 g/kg) group. (b): Percentage apoptosis in sections of hippocampal neuronal cells of rats. Results are representative of at least three independent experiments and expressed as the mean ± *SD*. Model group versus control group, **p < .01; high‐dose PLSO group versus model group, ^##^ *p* < .01; and positive group versus model group, ^###^ *p* < .001. (c): Effect of PLSO on the protein expression of Bax and Bcl‐2

Furthermore, the apoptosis rate was measured (Figure 8). Compared with the control group, the model group had a higher apoptosis rate, and the difference was very significant (*p* < .01). The positive group had a lower apoptosis rate than that of the model group, and the difference was very significant (*p* < .001). Compared with the model group, the apoptosis rate of the high‐dose PLSO group was low, and the difference was very significant (*p* < .01).

*Paeonia ludlowii* seed oil could inhibit expression of proinflammatory factors, thereby affecting the expression and phosphorylation of p65, reducing Bax content, and increasing the Bcl‐2 content. In this way, PLSO reduced damage to neuronal cells and apoptosis.

## 4 DISCUSSION

The drugs used for AD treatment can only relieve the symptoms of patients, but cannot slow the progression or cure AD (Lee et al., 2015). To discover and develop more efficacious and less toxic novel anti‐AD drugs or nutrient supplements, we used *P. ludlowii* seeds as raw materials to obtain PLSO through cold pressing. We established an AD model in rats to evaluate the anti‐AD efficacy of PLSO and compared it with that of donepezil (a classic drug for AD treatment).

We demonstrated that PLSO improved cognitive function significantly in AD rats by shortening the escape latency (Figure 1) and increasing the dwelling time on platform quadrants (Figure 2) compared with that of AD rats treated with physiologic saline. To study the specific effect of PLSO on AD, we used IHC detection of a microglia marker (IBA1) and astrocyte marker (GAFP) in the hippocampus (Figure 6). Next, we conducted RT‐qPCR detection of inflammatory factors (TNF‐α, IL‐1β, IL‐6) in hippocampal tissue (Figure 4). The action of proinflammatory factors affects the antioxidant capacity of cells. We measured expression of MDA, SOD, GSH, and ACHE, which are related to oxidation (Figure 5). PLSO could inhibit activation of microglia and astrocytes and expression of proinflammatory factors, thereby affecting the antioxidant capacity of cells. PLSO mediates expression of proinflammatory factors and then activates the NF‐κB signaling pathway, thereby affecting apoptosis and reducing damage to neuronal cells. To further study PLSO, we undertook Western blotting for detection of proinflammatory factors and p65 in the NF‐κB signaling pathway.

Increasing evidence from basic research in animal models of AD emphasizes that the key factor of neuronal injury/loss is due to neuronal apoptosis (Crews et al., 2010). Apoptosis plays a critical part in the development of the nervous system and in many chronic neurodegenerative diseases (including AD).

Cells undergo apoptosis *via* two major pathways: intrinsic and extrinsic (Spencer et al., 2010). The former occurs in mitochondria and mainly affects the Bcl‐2 family and caspases (Cerioni et al., 2010; Putcha et al., 2002). The Bcl‐2 family consists of pro‐apoptotic proteins (Bax, Bad, Bak) and anti‐apoptotic proteins (Bcl‐2, Bcl‐xL). The ratio of pro‐apoptosis proteins to anti‐apoptosis proteins directly determines the degree of opening of various channels in the mitochondrial outer membrane and regulation of apoptosis (Danial et al., 2003).

Bcl‐2 and Bax proteins have crucial roles in signal transduction during apoptosis. We showed that neuronal apoptosis plays a key part in AD pathogenesis and that PLSO inhibited neuronal apoptosis significantly (Figure 8b).

mRNA expression in rat neuronal tissue and protein expression of Bax and Bcl‐2 in PC12 cells was also analyzed. We found that PLSO could inhibit expression of proinflammatory factors, which affected the expression and the phosphorylation of p65. PLSO could downregulate expression of Bax protein and upregulate expression of Bcl‐2 protein to reduce damage to neurons and reduce the rate of apoptosis (Figure 8c).

These results suggested that PLSO could improve cognitive function significantly and prevent the neuronal injury induced through inhibiting apoptosis of neuronal cells in the hippocampus. PLSO could be even more efficacious against AD than donepezil in our AD model.

Liu and colleagues reported that the seed coat extract of peony could improve cognition defects, and they determined its active constituents (Liu et al., 2020). However, it is not known which type of drug component/molecule type caused the protective effect of PLSO upon rat neurons, or the related cell experiments, toxicity testing, and concentration gradients that need to be investigated further. However, the effects of PLSO on expression of Bax and Bcl‐2 suggest that the anti‐AD effect of PLSO may also be related to mitochondrial permeability, cytC, and the phosphoinositide 3‐kinase/protein kinase B signaling pathway. Therefore, further studies are needed to ascertain the mechanistic action of PLSO on the associated molecular pathways of neuronal apoptosis in the neurodegeneration seen in AD.

Although this new finding is promising, PLSO cannot be used as AD treatment. Animal testing is the first step in demonstrating its therapeutic potential. Human diseases are complex, and it is uncertain at which dose PLSO can be employed safely and efficaciously.

This study provides a scientific basis for the research and development of AD food products, which is helpful to reduce the use of a large number of AD drugs, promote the physiological health of Chinese people, improve people's quality of life, and reduce the medical burden related to AD to a certain extent.

## 5 CONCLUSIONS

We demonstrated that PLSO improved cognitive function significantly by inhibiting apoptosis of hippocampal neuronal cells. High‐dose PLSO could inhibit expression of proinflammatory cells by inhibiting activation of glial cells. Secretion of inflammatory factors causes production of free radicals and other substances, which leads to a reduction in the antioxidant capacity of cells, and PLSO could enhance the antioxidant capacity of cells.

*Paeonia ludlowii* seed oil was even more efficacious against AD than donepezil in our AD system. Furthermore, PLSO could downregulate expression of Bax protein and, simultaneously, upregulate expression of Bcl‐2 protein, by inhibiting activation of the NF‐κB pathway. Therefore, PLSO could be developed as a novel drug or nutrient supplement for the prevention and/or treatment of AD.

## CONFLICT OF INTEREST

The authors declare no conflicts of interest.
